# A myostatin-CCL20–CCR6 axis regulates Th17 cell recruitment to inflamed joints in experimental arthritis

**DOI:** 10.1038/s41598-021-93599-6

**Published:** 2021-07-08

**Authors:** Michelle Fennen, Toni Weinhage, Vanessa Kracke, Johanna Intemann, Georg Varga, Corinna Wehmeyer, Dirk Foell, Adelheid Korb-Pap, Thomas Pap, Berno Dankbar

**Affiliations:** 1 Institute of Musculoskeletal Medicine, University Hospital Muenster, Albert-Schweitzer-Campus 1, Bldg. D3, 48149 Muenster, Germany; 2 Department of Pediatric Rheumatology and Immunology, University Hospital Muenster, Muenster, Germany

**Keywords:** Rheumatoid arthritis, Chemokines, Chronic inflammation

## Abstract

The interactions of fibroblast-like synoviocyte (FLS)-derived pro-inflammatory cytokines/chemokines and immune cells support the recruitment and activation of inflammatory cells in RA. Here, we show for the first time that the classical myokine myostatin (GDF-8) is involved in the recruitment of Th17 cells to inflammatory sites thereby regulating joint inflammation in a mouse model of TNFalpha-mediated chronic arthritis. Mechanistically, myostatin-deficiency leads to decreased levels of the chemokine CCL20 which is associated with less infiltration of Th17 cells into the inflamed joints. In vitro, myostatin alone or in combination with IL-17A enhances the secretion of CCL20 by FLS whereas myostatin-deficiency reduces CCL20 secretion, associated with an altered transmigration of Th17 cells. Thus, the communication between activated FLS and Th17 cells through myostatin and IL-17A may likely contribute to a vicious cycle of inflammation, accounting for the persistence of joint inflammation in chronic arthritis. Blockade of the CCL20–CCR6 axis by inhibition of myostatin may, therefore, be a promising treatment option for chronic inflammatory diseases such as arthritis.

## Introduction

Rheumatoid arthritis (RA) is a severe systemic inflammatory and chronic autoimmune disease affecting the skeletal system and is characterized by a massive inflammation of synovial joints followed by bone and cartilage destruction^1,2^. During disease development, inflammation of the synovium promotes the transformation of fibroblast-like synoviocytes (FLS), which become activated and transform into a “tumor-like” phenotype^3,4^. Activated FLS promote disease severity by secreting various growth factors, pro-inflammatory cytokines and chemokines, driving the intra- and intercellular communication between FLS, immune cells, and osteoclasts (OC)^5–8^.

The complex interaction of FLS-derived pro-inflammatory cytokines/chemokines and immune cells support the recruitment and activation of innate and adaptive inflammatory cells like T cells, B cells, neutrophils, monocytes/macrophages as well as dendritic cells, which migrate from the blood stream into the affected joints and contribute to RA progression^5,7,9–13^. During disease progression, one of the most important processes is the invasion of T-cells, mainly CD4^+^ Th cells and CD8^+^ Tc cells^10,14^.

In this context, we have recently shown that beside known inflammatory modulators, FLS of RA patients and of arthritic mice display increased expression of myostatin (GDF-8) which belongs to the transforming growth factor-β (TGF-β) superfamily and is known to be a regulator of skeletal muscle growth and regeneration^15,16^. We could further demonstrate that myostatin was significantly involved in inflammatory bone loss and its deletion or pharmacological inhibition diminished joint inflammation and destruction in various mouse models of arthritis^17^. These data together with reduced inflammation upon blockade of myostatin in mice with chronic kidney disease (CKD)^18^ point to a general role of myostatin in the regulation of chronic inflammation.

Therefore, the aim of this study was to elucidate whether FLS-derived myostatin is involved in the recruitment of immune cells to the inflamed tissues and thereby contributes to the persistence of joint inflammation in a mouse model of TNFα-mediated chronic inflammation.

## Results

### Myostatin-deficiency leads to less inflammation in the hTNFtg mouse model of arthritis

In this study, we used myostatin-deficient arthritic (Mstn^−/−^ hTNFtg) mice to investigate the role of myostatin in regulating inflammation during arthritis development^17^. The hTNFtg mouse model is a well-established arthritis model in which a constitutive overexpression of TNFα is sufficient to induce a spontaneous chronic and severe rheumatoid arthritis phenotype, characterized by synovial inflammation and pannus formation, cartilage damage, and bone destruction^19^.

**Figure 1:**
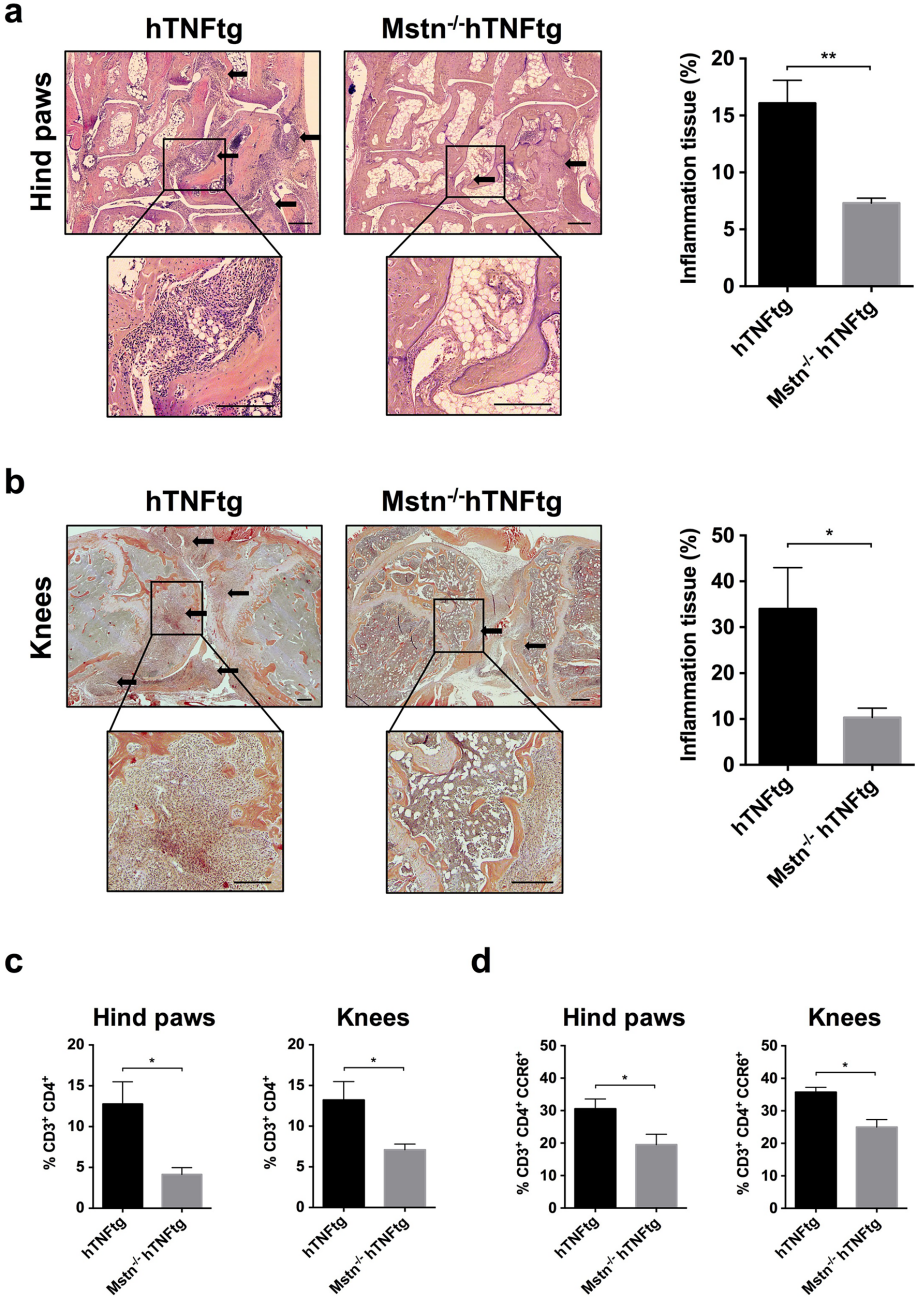
Less inflammation and infiltration of Th17 cells in joints of myostatin-deficient arthritic mice. Hematoxylin/Eosin staining of joint sections of hind paws and knees and analysis of Th cell populations in hind paw and knee joints of hTNFtg and Mstn^−/−^ hTNFtg mice by flow cytometry. (**a**) Representative images of hind paws and corresponding quantification of inflammation tissue. (**b**) Representative images of knee joints and corresponding quantification of inflammation tissue. Scale bars = 200 μm, arrows indicate inflammation tissue. Areas of inflammation tissue were evaluated by quantitative histomorphometric analysis. Data represent means ± SEM (n = 5 hind paws, n = 3 knees, Mann–Whitney *U* test, *p ≤ 0.05, **p ≤ 0.01). (**c**) Percentage of infiltrated CD4^+^ Th cells among the CD3^+^ T cell population in hTNFtg and Mstn^−/−^ hTNFtg mice. Data represent means ± SEM (hind paws: hTNFtg n = 7, Mstn^−/−^ hTNFtg n = 5; knees: hTNFtg n = 6, Mstn^−/−^ hTNFtg n = 5, Mann–Whitney *U* Test, *p ≤ 0.05). (**d**) Percentage of infiltrated CD3^+^ CD4^+^ Th cells expressing CCR6 in hTNFtg and Mstn^−/−^ hTNFtg mice. Data represent means ± SEM (hind paws: hTNFtg n = 5, Mstn^−/−^ hTNFtg n = 5; knees: hTNFtg n = 4, Mstn^−/−^ hTNFtg n = 5, Mann–Whitney *U* Test, *p ≤ 0.05).

In order to initially evaluate severity of inflammation, we performed histological stainings of hind paws and knees from arthritic hTNFtg mice. Subsequent analysis of these mice revealed severe inflammation with prominent synovial hyperplasia and marked infiltration of synovial tissues with inflammatory cells, which appeared to be less in Mstn^−/−^ hTNFtg mice (Fig. 1a,b). Quantitative histomorphometric analysis showed considerably less joint inflammation by about 50% in hind paws and about 70% in knees of Mstn^−/−^ hTNFtg compared to hTNFtg mice (Fig. 1a,b), substantiating an important regulatory role of myostatin in inflammation in arthritis.

### Reduced infiltration of Th17 cells in joints of myostatin-deficient hTNFtg mice

Since we observed less joint inflammation in myostatin-deficient mice, we wondered whether immune cell infiltration was reduced as well. When compared to uninflamed WT joints, analyses of CD45^+^ cells (leukocytes) demonstrated a high infiltration of various immune cells into the arthritic hind paw and knee joints of hTNFtg mice. Interestingly, Mstn^−/−^ hTNFtg mice showed, although not significant, a tendency towards less immune cell infiltration (hind paws: 19.06% versus 14.28%; knees: 20.98% versus 18.48%, respectively) (Supplementary Fig. S1a).

To analyse in more detail the effects of myostatin on different immune cell subpopulations in vivo, the cellular distribution of myeloid lineage cells, B cells and T cells in arthritic joints were assessed. To this end, we isolated joint cells from hind paws and knees and evaluated the differences in immune cell populations between hTNFtg and Mstn^−/−^ hTNFtg mice by FACS.

At first, we observed no obvious differences in the profile of main immune cell populations such as myeloid cells, B and T cells between the hTNFtg and Mstn^−/−^ hTNFtg mice (Supplementary Fig. S1a). Furthermore, subdivision of the myeloid cell population revealed again no differences in synovial tissue infiltration with neutrophils, dendritic cells, monocytes and macrophages (Supplementary Fig. S1b). Strikingly, further subdivision and analysis of CD3^+^ T cells demonstrated that the proportion of CD3^+^ CD4^+^ T helper cells (Th cells) was significantly reduced in hind paws (about 68%) as well as in knees (about 46%) of Mstn^−/−^ hTNFtg mice (hind paws: hTNFtg 12.7 ± 7.2% > Mstn^−/−^ hTNFtg 4.1 ± 1.8%; knees: hTNFtg 13.2 ± 5.5% > Mstn^−/−^ hTNFtg: 7.1 ± 1.5%), pointing to a considerable role of myostatin in Th cell recruitment (Fig. 1c).

Besides Th1, Th2 and Treg (regulatory) cells, Th17 cells and its secreted cytokine IL-17A have been shown to play a central role in the pathogenesis of various immune-mediated inflammatory diseases such as psoriasis, rheumatoid arthritis, multiple sclerosis and inflammatory bowel disease^14,20,21^. Since among the Th cell subsets only Th17 cells express CCR6, a chemokine receptor which is important for their recruitment into the inflamed tissue^5,22^, we quantified CCR6-expressing Th cells to evaluate whether synovial infiltration of Th17 cells was affected by the lack of myostatin. Interestingly, the proportion of CCR6-positive CD3^+^ CD4^+^ Th cells was also reduced in in the hind paws of Mstn^−/−^ hTNFtg mice (hTNFtg: 30.5 ± 6.7% > Mstn^−/−^ hTNFtg: 19.5 ± 7.1%) as well as in the knees (hTNFtg: 35.7 ± 3.0% > Mstn^−/−^ hTNFtg: 25.0 ± 5.1%), suggesting less recruitment of Th17 cells to the arthritic joints of myostatin-deficient hTNFtg mice (Fig. 1d). Similar numbers of Th17 cells in the lymph node and spleen of hTNFtg and Mstn^−/−^ hTNFtg mice further confirm the assumption that the reduced Th17 cell number in the joints of myostatin-deficient mice is not caused by a general reduction but rather by a reduced recruitment of Th17 cells (Supplementary Fig. S2).

Taken together, myostatin-deficiency in arthritic mice diminished the recruitment of Th17 cells to affected joints but did not influence the infiltration of myeloid and B cells.

### Myostatin-mediated increase in CCL20 secretion by FLS

In order to investigate by which mechanism myostatin affects the recruitment of Th17 cells, we analysed the secretion of the chemokine CCL20 by FLS, which has been described to be the sole ligand of the receptor CCR6^23,24^ and to be selective for the recruitment of Th17 cells in RA^5,25^. Because it is very likely that FLS-produced myostatin together with Th17 cell-produced IL-17 create an environment to further promote the recruitment of Th17 cells, we stimulated FLS with myostatin alone and additionally in combination with IL-17A.

**Figure 2:**
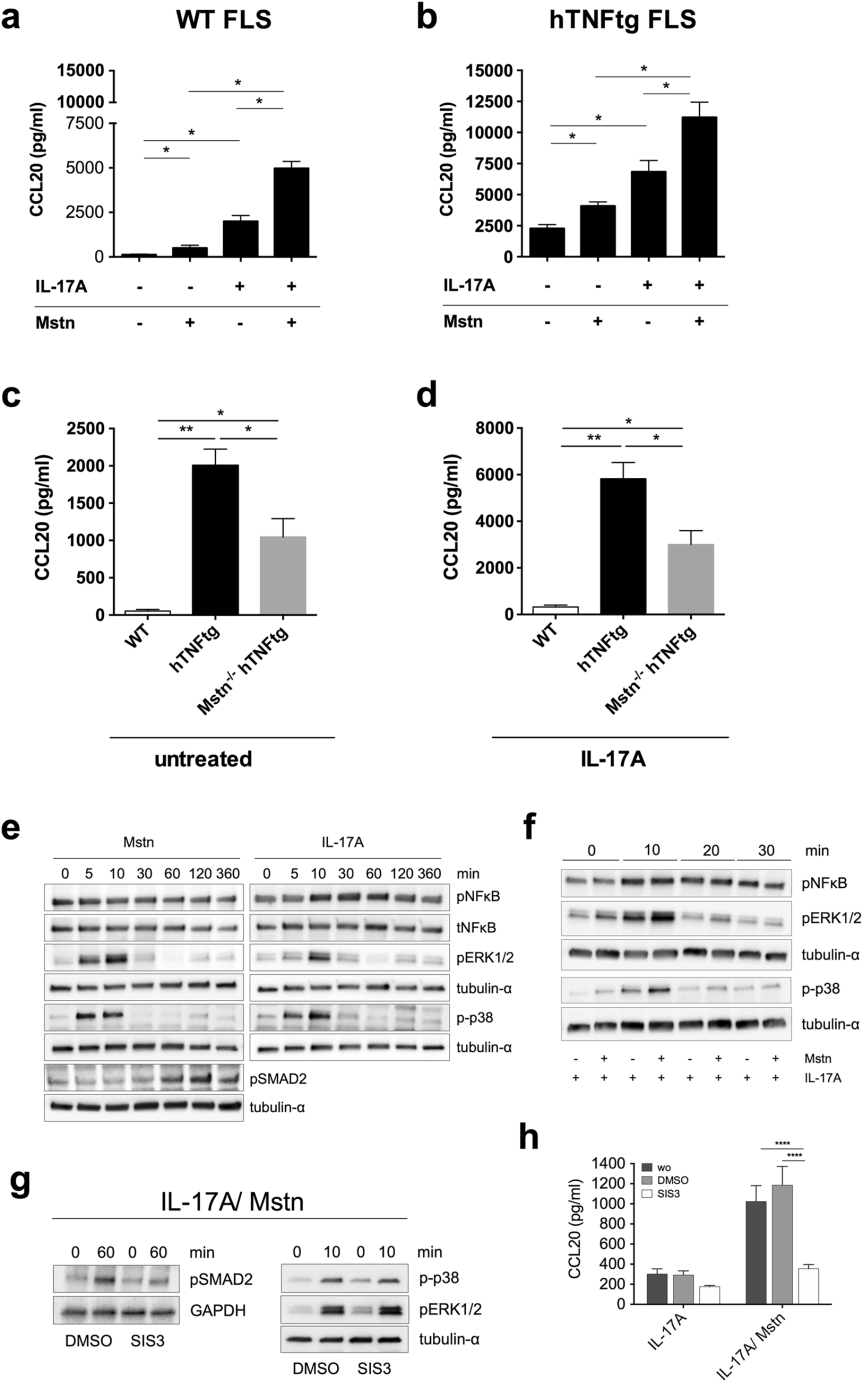
Myostatin-mediated SMAD-dependent increase in CCL20 secretion by FLS. Regulation of CCL20 secretion by FLS and determination of involved signaling pathways. (**a**) WT and (**b**) hTNFtg FLS were stimulated with IL-17A (20 ng/ml) and Mstn (100 ng/ml) alone or in combination or left untreated for 48 h. CCL20 concentrations were measured in the supernatants by ELISA. Data represent means ± SEM (n = 4; Mann–Whitney *U* test, *p ≤ 0.05). (**c**) FLS from WT, hTNFtg and Mstn^−/−^ hTNFtg mice were left untreated or (**d**) stimulated with IL-17A (20 ng/ml) for 48 h. CCL20 concentrations were measured in the supernatants by ELISA. Data represent means ± SEM (WT n = 3, hTNFtg n = 7, Mstn^−/−^ hTNFtg n = 6, Mann–Whitney *U* Test, *p ≤ 0.05, **p ≤ 0.01). (**e**) Phosphorylation of NFκB, ERK, p38 and SMAD2 in FLS after stimulation with myostatin (100 ng/ml) or IL-17A (20 ng/ml) for the indicated time-points. Phosphorylation of SMAD2 was not induced by IL-17A. α-tubulin served as loading control. Representative blots are depicted (n = 4). (**f**) Phosphorylation of NFκB, ERK and p38 in FLS stimulated either with IL-17A (20 ng/ml) alone or in combination with myostatin (100 ng/ml) for the indicated time-points. Representative blots are shown (n = 4). (**g**) Specific inhibition of SMAD2 signaling by the inhibitor SIS3 without affecting MAPK activation. DMSO was used as a control. Representative blots are shown (n = 4). (**h**) Effect of SMAD2 inhibition on the secretion of CCL20 by IL-17A- or IL-17A/Myostatin-stimulated FLS. CCL20 levels were measured in supernatants of FLS after 48 h of stimulation by ELISA. Data represent means ± SEM (n = 4; two-way ANOVA and multiple comparison test, ***p ≤ 0.001). Full-length blots are presented in Supplementary information.

Indeed, concomitant stimulation of FLS with IL-17A and myostatin revealed that myostatin was able to increase the IL-17A-mediated secretion of CCL20 by WT as well as by hTNFtg FLS. Treatment of WT FLS led to a significantly enhanced release of CCL20 by IL-17A (18-fold, 1998.1 ± 665.0 pg/ml) compared to untreated control cells (118.2 ± 54.5 pg/ml). Notably, the release of CCL20 was further enhanced by about 2.6-fold in the presence of IL-17 and myostatin (4969.3 ± 789.0 pg/ml, Fig. 2a).

Likewise, the secretion of CCL20 by hTNFtg FLS was increased by IL-17A and could be further enhanced by IL-17A together with myostatin (IL-17A: 6835.9 ± 1813.8 pg/ml; IL-17A/Mstn: 11,219.2 ± 2430.5 pg/ml, 1.7-fold induction, p ≤ 0.05, Fig. 2b). Notably, myostatin alone was also able to increase the low basal secretion of CCL20 in comparison to untreated controls by 4.2-fold in WT FLS and 1.8-fold in hTNFtg FLS (Fig. 2a,b). Taken together, myostatin appears to be an important regulator of CCL20 secretion by FLS.

In consideration of the fact, that myostatin is expressed by FLS^17^, we further investigated whether the increase in CCL20 secretion is mediated by an autocrine effect. Comparison of FLS obtained from WT, hTNFtg and Mstn^−/−^ hTNFtg mice revealed that arthritic FLS had significantly higher basal CCL20 levels, which were around 37-fold and 19-fold higher than in WT FLS, respectively (WT FLS: 53.7 ± 35.4 pg/ml, hTNFtg FLS: 2004.2 ± 583.2 pg/ml, Mstn^−/−^ hTNFtg FLS: 1047.0 ± 600.8 pg/ml; Fig. 2c). As expected, stimulation with IL-17A led to a remarkably enhanced CCL20 secretion, in which again WT FLS showed a significantly lower IL-17A-mediated CCL20 secretion compared to hTNFtg and Mstn^−/−^ hTNFtg FLS (WT FLS: 323.8 ± 135.1 pg/ml, hTNFtg FLS: 5825.6 ± 1836.9 pg/ml, Mstn^−/−^ hTNFtg FLS: 3001.4 ± 1462.9 pg/ml; Fig. 2d). Most importantly, FLS from myostatin-deficient arthritic mice showed significantly reduced basal as well as stimulated levels of CCL20 (about 48%) compared to corresponding hTNFtg FLS, confirming an autocrine effect of myostatin on CCL20 secretion (Fig. 2c,d).

In summary, the data clearly demonstrated that myostatin is able to increase basal as well as IL-17A-mediated secretion of CCL20 by WT and hTNFtg FLS in an autocrine manner.

### Enhanced CCL20 secretion by myostatin depends on SMAD but not on MAPK signaling

In order to clarify by which signaling pathways myostatin enhanced the secretion of CCL20 by FLS, we analysed the activation of NFκB as well as the MAPK ERK1/2 and p38. Phosphorylation of ERK1/2 and p38 occurred five minutes, activation of SMAD signaling 60 min after stimulation with myostatin whereas activation of NFκB could not be detected. Stimulation of FLS with IL-17A led to the phosphorylation of ERK1/2 and p38 five to ten minutes after stimulation whereas phosphorylation of NFκB was detected after 10 min (Fig. 2e).

Moreover, western blot analysis indicated that myostatin was able to enhance IL-17A-mediated ERK1/2 and p38 MAPK activation whereas the activation of NF-κB was not influenced (Fig. 2f). To differentiate whether the activation of the MAPK pathway or activation of the SMAD signaling is the cause of increased CCL20 secretion by FLS, SMAD signaling was inhibited during simultaneous stimulation with myostatin and IL-17A. As expected, the SMAD inhibitor selectively inhibited the activation of SMAD2 upon stimulation with IL-17A/myostatin with no effects on the MAPK pathways (Fig. 2g). Interestingly, analysis of corresponding CCL20 secretion revealed a highly significant reduction in CCL20 levels after inhibition of SMAD2 activation (Fig. 2h), indicating that the myostatin-mediated increase in CCL20 secretion depends exclusively on activation of the SMAD pathway.

### Decreased levels of CCL20 in hind paws of myostatin-deficient hTNFtg mice

Based on the reduced secretion of CCL20 by arthritic FLS lacking myostatin in vitro (Fig. 2a–d), we further investigated whether reduced CCL20 levels were detectable within the tarsal joints of myostatin-deficient arthritic mice as well.

**Figure 3:**
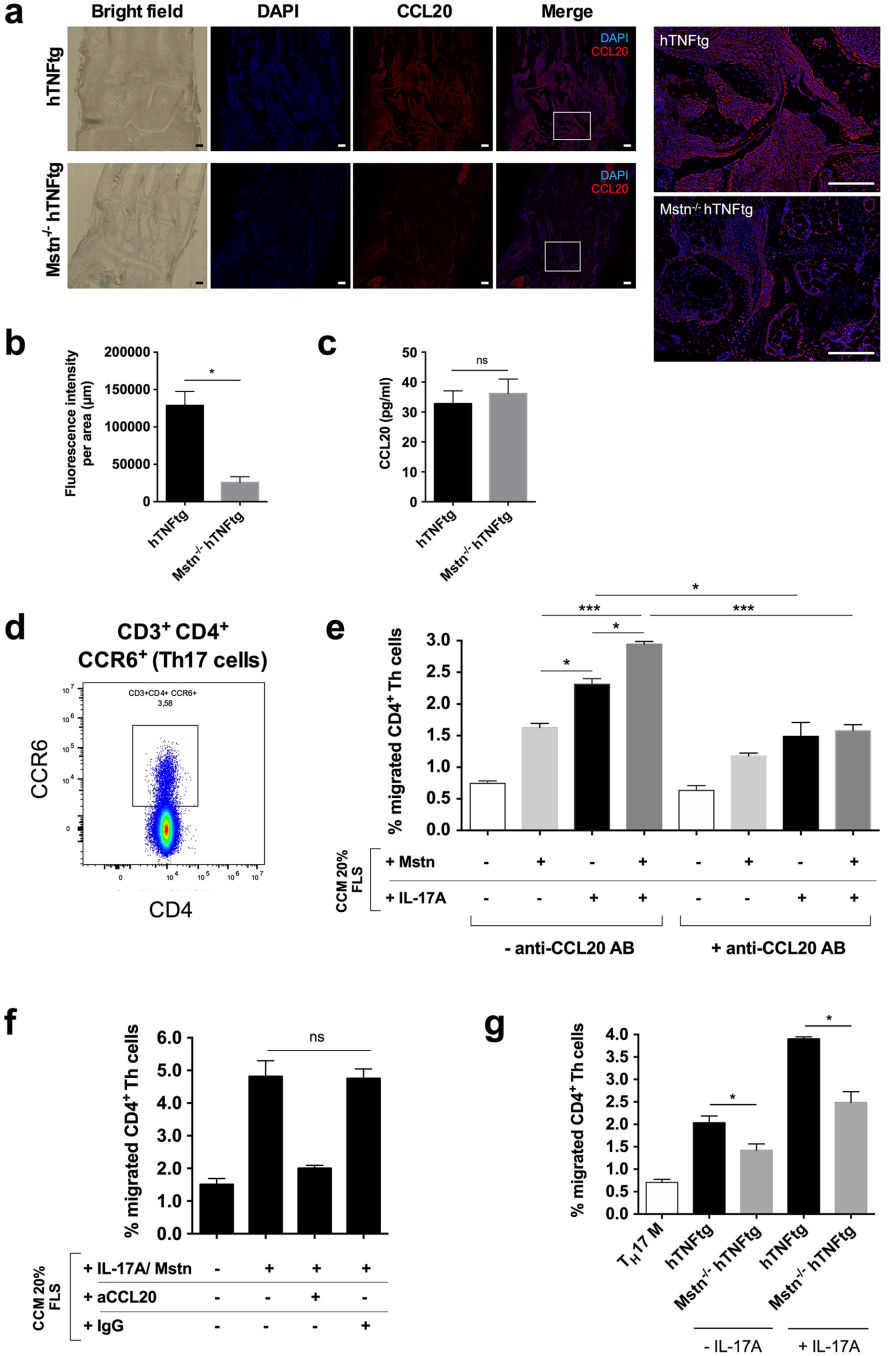
Decreased levels of CCL20 in joints of myostatin-deficient arthritic mice and regulation of Th17 cell transmigration by FLS-secreted CCL20. Immunohistochemical staining of CCL20 in tarsal joint sections of arthritic mice and CCL20-mediated transmigration of Th cells in vitro. (**a**) Representative images of bright field (left) and fluorescence-stained hind paw sections from hTNFtg and Mstn^−/−^ hTNFtg mice (nuclear staining: DAPI—blue, CCL20: Alexa 546—red) and a higher magnification of stained areas are depicted. Scale bars = 200 μm. (**b**) Fluorescence intensity of CCL20 stainings within the ROI (normalised to IgG control-stained sections) of hTNFtg and Mstn^−/−^ hTNFtg mice. Data represent means ± SEM (hTNFtg: n = 3, Mstn^−/−^ hTNFtg n = 5, Mann–Whitney *U* Test, *p ≤ 0.05). (**c**) CCL20 serum levels of hTNFtg and Mstn^−/−^ hTNFtg mice. Data represent means ± SEM (hTNFtg: n = 12, Mstn^−/−^ hTNFtg n = 10, Mann–Whitney *U* Test). (**d**) Proportion of CCR6-expressing cells in LN-derived CD4^+^ Th cells (%). (**e**) CD4^+^ Th cell transmigration rate towards 20% conditioned culture medium (CCM) of myostatin-stimulated, IL-17A-stimulated, or simultaneously stimulated FLS either with or without anti-CCL20 antibody treatment. Data represent means ± SEM (n = 4, two-way ANOVA and multiple comparison test, *p ≤ 0.05, **p ≤ 0.01, ***p ≤ 0.001). (**f**) Specificity of CCL20 inhibition on CD4^+^ Th cell transmigration by the anti-CCL20 antibody. Data represent means ± SEM (n = 4, Wilcoxon Test). (**g**) Effect of CCM of hTNFtg and Mstn^−/−^ hTNFtg FLS stimulated with or without IL-17A. Data represent means ± SEM (hTNFtg and Mstn^−/−^ hTNFtg n = 4, each, Mann–Whitney *U* Test, *p ≤ 0.05). T_H_17 medium (T_H_17 M) without CCM served as a negative control.

Indeed, fluorescence staining of hind paw sections showed clearly reduced CCL20 staining in the tarsal joints of Mstn^−/−^ hTNFtg mice compared to hTNFtg mice (Fig. 3a). Quantification of the fluorescence intensity of CCL20 staining revealed about 5 times lower levels of CCL20 in tarsal joints from myostatin-deficient hTNFtg mice compared to hTNFtg mice (Fig. 3b), probably reflecting the reduced secretion of CCL20 by myostatin-deficient arthritic FLS. In contrast, serum levels of CCL20 were not affected by the loss of myostatin, suggesting a tissue-specific effect of myostatin on CCL20 secretion restricted to the joint cells within the inflamed synovial tissues (Fig. 3c).

### Elevated CD4^+^ Th cell migration due to increased secretion of CCL20 by FLS

Finally, we examined whether the increased CCL20 secretion by FLS induced by myostatin and/or IL-17 facilitates an increased Th cell (CD4^+^) migration in vitro. Since it has been demonstrated that CCL20 is mainly involved in the recruitment of CCR6^+^ Th cells^5,22,25^, we first evaluated the proportion of CCR6^+^ Th cells in the CD3^+^ CD4^+^ Th cell subpopulation isolated from lymph nodes. Approximately 3–4% of the purified cells were positive for CCR6, displaying the subpopulation of Th cells that could be recruited by CCL20 (Fig. 3d).

In vitro chemotaxis/transmigration assays showed that the addition of 20% conditioned culture medium (CCM) from both myostatin- and IL-17A-treated FLS significantly increased CCR6^+^ Th cell migration at which IL-17A treatment was more effective than treatment with myostatin (2.31 ± 0.17% vs. 1.63 ± 0.11%, Fig. 3e). Most interestingly, CCM of FLS concomitantly stimulated with myostatin and IL-17A further increased CCR6^+^ Th cell migration compared to CCM of separately stimulated FLS (2.95 ± 0.086%, Fig. 3e), reflecting about 80% of Th cells which were recruitable by CCL20 at all. In order to confirm that the stimulatory effects of CCM on Th cell migration was in fact mediated by CCL20, we used anti-CCL20 neutralizing antibody to determine inhibitory effects on CCR6^+^ Th cell migration. Th cell migration induced by CMM of IL-17A-as well as myostatin/IL-17A-stimulated FLS was effectively inhibited by treatment with an anti-CCL20 neutralizing AB (1.49 ± 0.44% and 1.58 ± 0.19%, respectively, Fig. 3e,f). Absent effects of the corresponding IgG isotype control on CCR6^+^ Th cell migration confirmed the specificity of the CCL20 inhibition (Fig. 3f). Importantly, CCM of FLS from myostatin-deficient arthritic mice regardless whether they were stimulated with IL-17A or not induced significantly less CCR6^+^ Th cell migration (about 30% and 36%, respectively) compared to corresponding CMM of hTNFtg FLS (Fig. 3g), explaining the reduced recruitment of Th cells to the arthritic joints of myostatin-deficient hTNFtg mice.

Collectively, the results indicate that myostatin-mediated increased expression of CCL20 by FLS is involved in the trafficking of CD3^+^CD4^+^CCR6^+^ Th cells, most likely Th17 cells, towards the inflammation tissues in arthritic joints.

## Discussion

It is commonly accepted that the interaction of immune cells and activated FLS plays a pivotal role in the development and persistence of chronic inflammatory arthritis by secreting a panel of inflammatory cytokines^3,6–9^ and chemokines^26–29^. In this context, we have recently shown that beside known inflammatory modulators, arthritic FLS display increased expression of the myokine myostatin, which is regulated by key cytokines of RA such as TNFα, IL-1 and notably most strongly IL-17. Moreover, deletion or pharmacological inhibition of myostatin diminished joint pathology in various mouse models of arthritis^17^.

Based on these findings, the aim of this study was to elucidate whether FLS-derived myostatin is involved in the recruitment of immune cells to the inflamed tissues thereby contributing to the persistence of joint inflammation in a chronic TNF-a-mediated arthritis mouse model.

Initial analysis of hind paws revealed substantially less joint inflammation in myostatin-deficient arthritic mice and this was accompanied by an overall reduced immune cell infiltration. Interestingly, detailed analysis of the immune cell composition in arthritic joints revealed a tremendous reduction exclusively in the Th cell population whereas infiltration of joint tissues with neutrophils, dendritic cells, monocytes, macrophages and B cells was not significantly affected by the lack of myostatin, pointing to myostatin being particularly involved in Th cell recruitment.

Interestingly, further subdivision of the Th cell population revealed that the proportion of Th cells expressing CCR6, a chemokine receptor known to be expressed by Th17 cells, was significantly reduced in arthritic joints of Mstn^−/−^ hTNFtg mice compared to hTNFtg joints as well. In consideration of these finding we suppose that myostatin promotes the recruitment of Th17 cells into the inflamed joints of arthritic mice.

This assumption is supported by Pene and colleagues, who showed that CCR6 cell surface expression is mainly restricted to tissue-infiltrating Th17 and not to Th1 or Th2 cells isolated from inflamed tissues of human patients^24^. Additionally, Saini et al. described that IL-17A is mainly produced by CD3^+^CD4^+^ Th cells, which are additionally positive for CCR6 revealing that CD3^+^CD4^+^CCR6^+^ T cells can be considered as Th17 cells^30^.

IL-17 produced by Th17 cells is involved in a proinflammatory feedback loop with synovial fibroblasts supporting pro-inflammatory changes in the synovium and promoting inflammation due to the stimulatory effect on FLS to secrete pro-inflammatory cytokines, chemokines as well as osteoclast-promoting factors^13,14,20,31–33^. Indeed, higher numbers of Th17 cells and higher expression levels of IL-17 have been consistently detected in arthritis patients and various studies supported a positive correlation with disease activity^31–35^. In this regard, it is important to know that increased Th17 cell populations in inflamed joints are supposed to be caused by enhanced migration of CCR6^+^ Th17 cells dependent on elevated MIP-3alpha (CCL20) levels^5,25,36^. Since IL-17 besides myostatin has been shown to induce the expression of CCL20 in RA-FLS, activated FLS in the inflamed synovium may further recruit Th17 cells through increased CCL20 production^25,37,38^. Thus, it is very likely that upon communication between Th17 cells and FLS, Th17 cell-produced IL-17 collectively with FLS-produced cytokines and chemokines form a milieu to cross-regulate the recruitment of Th17 cells.

Of prime importance, the CCL20–CCR6 axis plays a major role in RA in which an inappropriate activation of this chemokine network has been connected to disease aggravation^38^. CCL20, known to be the only ligand for CCR6, has been demonstrated to attract leukocytes, preferentially Th17 cells, to the inflamed tissue of RA affected joints and its expression has shown to be highly upregulated in synovial tissues of RA patients^5,25,37,39^. Therefore, we assumed that the impaired recruitment of CCR6^+^ Th17 cells to the joints of myostatin-deficient arthritic mice is based on lower synovial levels of CCL20. Indeed, myostatin was able to significantly enhance the basal as well as the IL-17A-mediated secretion of CCL20 in both WT FLS and stably activated FLS from hTNFtg mice via the SMAD pathway. Consistently, myostatin-deficiency leads to reduced secretion of CCL20 in arthritic FLS in vitro regardless of stimulation with IL-17A, strongly indicating that myostatin represents an autocrine regulator of CCL20 secretion by FLS. Accordingly, an autocrine role of myostatin has recently been established by our group, showing that OC differentiation in vitro was strongly diminished in myostatin-deficient bone marrow macrophages^17^. Comparative analyses of CCL20 expression in hTNFtg and myostatin-deficient hTNFtg mice revealed highly reduced levels of CCL20 in hind paws of Mstn^−/−^ hTNFtg by approximately 80% compared to those of hTNFtg mice strongly substantiating the assumption that myostatin constitutes an important regulator of CCL20 under inflammatory conditions in vivo as well.

Finally, functional analyses of CCL20 levels by transmigration assays showed that increased CCL20 secretion by stimulated FLS specifically mediates an increase in Th cell migration in vitro. In detail, conditioned medium from myostatin- or IL-17A-stimulated FLS highly promoted the transmigration of Th cells and was further increased by CCM of FLS concomitantly stimulated with IL-17A and myostatin, clearly indicating that CCM-induced Th cell migration depends on CCL20. Moreover, the fact that high levels of CCL20 in the hTNFtg FLS cultures were associated with a high Th cell migration rate, whereas lower levels of CCL20 observed by myostatin-deficient hTNFtg FLS were reflected by reduced migration of Th cells, leads to the conclusion that FLS-derived myostatin plays a key role in the trafficking of CD3^+^CD4^+^CCR6^+^ Th17 cells^5,24,30^, thereby supporting sustained chronic inflammation in hTNFtg mice.

One of the most important events in RA is the infiltration of T cells in the synovial compartment of the inflamed joint^10,14,40–42^ and there is plenty of evidence that among the CD4^+^ Th cells, the IL-17-producing Th17 cells, play a protruding role in RA pathogenesis. Increased Th17 cell populations and levels of IL-17A in joints of RA patients have been both associated with disease severity^5,24,25,41^. Even though initiation of arthritis in hTNFtg mice is not dependent on T and B cell activation^43^, we strongly assume that the presence of Th17 cells and its secreted factor IL-17A at later stages of arthritis is responsible for disease persistence and progression. This is supported by studies from us and others demonstrating that Th cells including Th17 cells are constantly detectable or even increased in the synovium of hTNFtg mice also at later disease stages^44–46^ and that adoptive transfer of Th cells augments disease severity in other arthritis mouse models^47,48^.

In conclusion, we could show for the first time that a classical myokine is involved in the recruitment of immune cells thereby regulating joint inflammation in a mouse model of chronic arthritis. Our data strongly support the hypothesis that myostatin regulates the recruitment of Th17 cells through increased levels of CCL20 in arthritic joint tissues, which subsequently leads to increased cytokine levels including IL-17. Since IL-17 strongly regulates myostatin expression in FLS^17^, it is likely that high levels of myostatin account for additionally CCL20 secretion, further supporting Th17 cell infiltration. Thus, the communication between activated FLS and Th17 cells through myostatin and IL-17A may lead to a vicious cycle of inflammation with perpetual Th17 cell infiltration, contributing to the persistence of joint inflammation in chronic arthritis. Blockade of myostatin could be therefore an interesting target for the treatment of chronic inflammatory diseases, to inhibit not only bone destruction but simultaneously inflammation.

Ultimately, beside its prominent role in arthritis, the CCL20–CCR6 axis has also been found to be implicated in a number of other autoimmune-inflammatory conditions including psoriasis, asthma and inflammatory bowel disease^21,38^. Thus, inhibition of CCL20–CCR6 interactions by myostatin blockade may be a promising treatment option for other autoimmune and inflammatory disorders as well.

## Materials and methods

### Animals

Myostatin-deficient arthritic mice were generated by crossing myostatin knockout (Mstn^−/−^) mice^16^ into human TNFα transgenic (hTNFtg) mice^19^, which develop a chronic inflammatory destructive arthritis^17^. Both mouse strains were kept on the C57BL/6J genetic background. Standard animal husbandry in accordance with the institutional guidelines was performed to generate cohorts of male and female mice. All animal procedures have been approved by the local ethics committee “Landesamt für Natur, Umwelt und Verbraucherschutz Nordrhein-Westfalen” (LANUV; 84.02.04.2015.A060) and were carried out in compliance with the ARRIVE guidelines.

### Histomorphometric analysis

Hind paws of mice at week 12 were fixed overnight in 4% formalin and then decalcified in EDTA. Paraffin-embedded sections were stained with hematoxylin–eosin for assessment of synovial inflammation. Quantification of inflammation was performed using a Zeiss Observer.Z1 and an image analysis system (Zeiss AxioVision 4.8. software; Carl Zeiss, Marburg, Germany).

### Isolation, culture and stimulation of FLS

Hind paws were dissected under sterile conditions and the skin, tendons, and toes were removed. Primary FLS were isolated from hind paws by enzymatic digestion with 1 mg/ml Collagenase IV (Worthington, Lakewood, USA). Isolated FLS were cultured in Dulbecco’s modified Eagle’s medium (DMEM) with high glucose (Sigma-Aldrich, Darmstadt, Germany) supplemented with 10% heat-inactivated FCS (Biochrom GmbH, Berlin, Germany). FLS were used in passages 3–5. FLS (5 × 10^4^ cells/ 24-well) were stimulated with murine myostatin (100 ng/ml), murine IL-17A (20 ng/ml) or with a combination of both (R&D Systems, Nordenstadt, Germany) for 48 h. Supernatants were collected and either used for assessment of CCL20 secretion or as conditioned medium (CM) in T-cell migration assays.

### Determination of CCL20 secretion

To quantify secretion of CCL20 by FLS, supernatants of unstimulated and stimulated FLS were subjected to ELISA analysis (R&D Systems). Individual steps were performed according to the manufacturer's instructions. CCL20 concentrations of individual supernatants were corrected for total protein content of corresponding FLS.

### Th cell isolation and transmigration assay

Primary murine CD4^+^ Th cells were isolated from spleen and assessor and proper axillary lymph nodes (LN), subiliac LN and popliteal LN behind the knee^49^ of both sides of euthanized mice using a MojoSort™ Mouse CD4 T Cell Isolation Kit (BioLegend, San Diego, USA) according to manufacturer´s instructions. The purity of isolated CD4^+^ Th cells was analysed using the CytoFLEX S flow cytometer, CytExpert Software (Beckman Coulter Life Sciences, Indianapolis, USA) and FlowJo 10.5.3 software (Ashland, Oregon, USA). 1 × 10^5^ living/single cells (Zombie NIR™ negative) were selected and gated by the expression in the lineage trace marker (LIN-FITC) and CD3 (CD3-BV 421™) to remove monocytes, neutrophils, granulocytes, macrophages, B-cells, and erythrocytes from the analysis. Finally, only CD3^+^ CD4^+^ Th cells were investigated, which were further analysed by the expression of the cell surface receptor CCR6 (CD196^+^). Th cells were kept on ice in RPMI-1640 medium supplemented with 2% heat-inactivated FCS, 1% l-Glutamine Solution (200 mM) and 1% MEM Non-essential Amino Acid Solution (Th cell medium). All media components were from Sigma-Aldrich, Darmstadt, Germany.

Cell transmigration was performed using a transwell system with a permeable polycarbonate membrane containing a 5 µm pore size (Corning, ThermoFischer Scientific, Schwerte, Germany). 5 × 10^5^ CD4^+^ Th cells in Th cell medium were placed in the upper well of the Corning™ Costar™ Transwell™ system. The lower well was filled with 600 µl Th cell medium as control or 20% conditioned culture medium (CMM) from stimulated FLS diluted in Th cell medium. Migration of CD4^+^ Th cells was blocked using a neutralizing rabbit anti-CCL20/MIP-3α antibody at a concentration of 8 µg/ml (Abcam, Berlin, Germany). For this purpose, the antibody was incubated for 2 h at 37 °C and 5% CO_2_ in Th cell medium with 20% CMM prior to the migration experiments. To control specificity of antibody blocking, a concentration-matched anti-rabbit IgG control was used (Cell signaling, Frankfurt a.M., Germany). Transmigration of CD4^+^ Th cells was performed for 4 h at 37 °C and 5% CO_2_ and the number of CD4^+^ Th cells migrated through the polycarbonate membrane was quantified and calculated as the proportion (%) of initially seeded cells.

### Immunoblotting

Total cell extracts of FLS were obtained using NP-40 buffer (150 mM sodium chloride, 1% NP-40, and 50 mM Tris–HCl (pH 8)) containing protease and phosphatase inhibitor cocktail (Sigma-Aldrich). Extracts were resolved by SDS–polyacrylamide gel electrophoresis (PAGE) and transferred to a PVDF membrane. The proteins were detected with appropriate antibodies using the ECL detection system (ThermoFischer Scientific, Schwerte, Germany). Antibodies against the following proteins were used: phospho-ERK, phospho-p38, phospho-NFκB, total NFκB, phospho-SMAD2 (all from Cell Signaling). All phospho-antibodies were used as 1:1000 dilutions. For the purpose of control, blots were stripped and re-probed for GAPDH (Cell Signaling) or α-tubulin (Sigma-Aldrich). Signals were detected by Fusion FX Western Blot Imager (Vilber Lourmat, Eberhardzell, Germany).

### Immunohistochemistry

Tissue samples from hind paws of hTNFtg and Mstn^−/−^/hTNFtg mice were fixed in 4% paraformaldehyde, decalcified in 10% EDTA/TBS, embedded into paraffin and sectioned into 5 μm slices. Antigen retrieval was performed using 0.05% Proteinase XXIV (Sigma-Aldrich) in a humid chamber for 8 min at 37 °C. After blocking with 2% BSA/TBS, CCL20 (MIP-3α) antibody (Abcam) was applied to the slides and incubated over night at 4 °C followed by a donkey anti-rabbit AF546 secondary antibody (ThermoFischer Scientific) for 90 min in the dark at RT. Concentration matched anti-rabbit IgG control (Cell signaling) was used as control. Finally, slides were stained with DAPI, fixed with fluoromount aqueous mounting medium (Sigma-Aldrich) and stored at 4 °C in the dark.

For expression analysis of CCL20 in joints of hTNFtg and Mstn^−/−^/hTNFtg mice, images of immunofluorescent stained sections were taken using a fully-motorized and automated IX83 inverted microscope (Olympus Europa SE & Co.KG, Hamburg, Germany). Quantification of CCL20 expression was performed using Olympus CellSens software (Olympus Europa SE & Co.KG). A ROI was defined around the tarsal joints and a threshold of fluorescence intensity was defined on anti-rabbit IgG control stained sections. Subsequently, the area of positive stained CCL20 was compared between Mstn^−/−^/hTNFtg and hTNFtg hind paws.

### Isolation of cells from knees and hind paws

Hind limbs were dissected and the skin, muscles and tendons were carefully removed from legs without damaging the bones and joints. Hind paws and opened knee joints were then digested with collagenase D (60 µg/ml, Roche) and 3U DNAse (Zymo Research, Irvine, USA) in RPMI (2% FCS) for 30 min at 37 °C. Hind paws and knee joints were rinsed with RPMI (2% FCS) and the obtained filtered cell suspension was stored on ice. The remaining tissue was then further digested with collagenase/dispase (60 µg/ml, Roche) and 3U DNase in RPMI (2% FCS) for 30 min at 37°. Afterwards, 15 μl EDTA (0.5 M, pH 8.0) was added to each tube and incubated for another 5 min at 37 °C. The recovered cell suspension was filtered, combined with the previously obtained cell suspension and centrifuged at 350 g for 4 min at RT. The supernatant was discarded and the cell pellet was treated with red blood cell lysis buffer for 4 min at RT. Subsequently, cells were washed with PBS, centrifuged, resuspended in PBS and used directly for cell surface marker staining and FACS analysis.

### Flow cytometry analysis

In order to determine immunophenotypical differences between WT, hTNFtg and Mstn^−/−^/hTNFtg mice, compensation for each antibody was performed on beads (BD™ CompBead Anti-Rat and Anti-Hamster Ig κ/Negative Control Compensation Particles Set, BD Biosciences, Frankling Lakes, USA; or VersaComp Antibody Capture Bead Kit, Beckmann Coulter Inc., Bree, USA) and single stained cells. To avoid unspecific binding of IgG’s to Fc-receptors, isolated cells were incubated with 1 μg anti-mouse CD16/32 antibody for 10 min at 4 °C in the dark. Anti-mouse antibody panels against cell surface antigens from the myeloid linage (CD45-APC/Cy7, CD11b-PerCP/Cy5.5, CD11c-PE/Cy7, Ly6C-PE, Ly6G-APC, MHC class II (I-A/I-E)-FITC and DAPI (living cells) or the lymphoid linage (CD45-APC, CD11b-PerCP/Cy5.5, CD19-APC/Cy7, CD3-PE/Cy7, CD4-FITC, CD8-PE, DAPI as well as CD196-PE/Dazzel were used for specific immune cell staining. All fluorescent-labeled antibodies were obtained from BioLegend. The immune cell composition of each tissue was analysed using the CytoFLEX S flow cytometer (Beckman Coulter Life Sciences) and CytExpert Software (Beckman Coulter Life Sciences) using defined gating strategies (Supplementary Fig. S3).

### Statistical analyses

Statistical analysis was performed using GraphPad Prism 7 software (Graph Pad Software Inc., San Diego, USA). If normal distribution was not valid, statistical significance was evaluated using the Mann–Whitney rank sum test for differences between two independent groups, and the Wilcoxon matched-pair signed rank test for comparison of differences within pairs. Statistical significance of overall differences between multiple groups was analyzed by the two-way ANOVA. If the test was significant, pairwise comparisons were done by a multiple comparison test. A P value of less than 0.05 was considered statistically significant.

## Supplementary Information

Supplementary Information.

## Data Availability

All study data are included in the article and supporting information.
