# Supplementary material for: A myostatin-CCL20–CCR6 axis regulates Th17 cell recruitment to inflamed joints in experimental arthritis
Source: Sci Rep. 2021 Jul 8;11:14145. doi: 10.1038/s41598-021-93599-6 (PMC8266846; doi:10.1038/s41598-021-93599-6)

A Myostatin-CCL20-CCR6 axis regulates Th17 cell recruitment to inflamed joints in experimental arthritis

Michelle Fennen[1], Toni Weinhage[2], Vanessa Kracke[1], Johanna Intemann[1], Georg Varga[2], Corinna Wehmeyer[1], Dirk Foell[2], Adelheid Korb-Pap[1], Thomas Pap[1], Berno Dankbar[1]

[1]Institute of Musculoskeletal Medicine, University Hospital Muenster, Muenster, Germany;

[2]Department of Pediatric Rheumatology and Immunology, University Hospital Muenster, Muenster, Germany

Supplemental figures

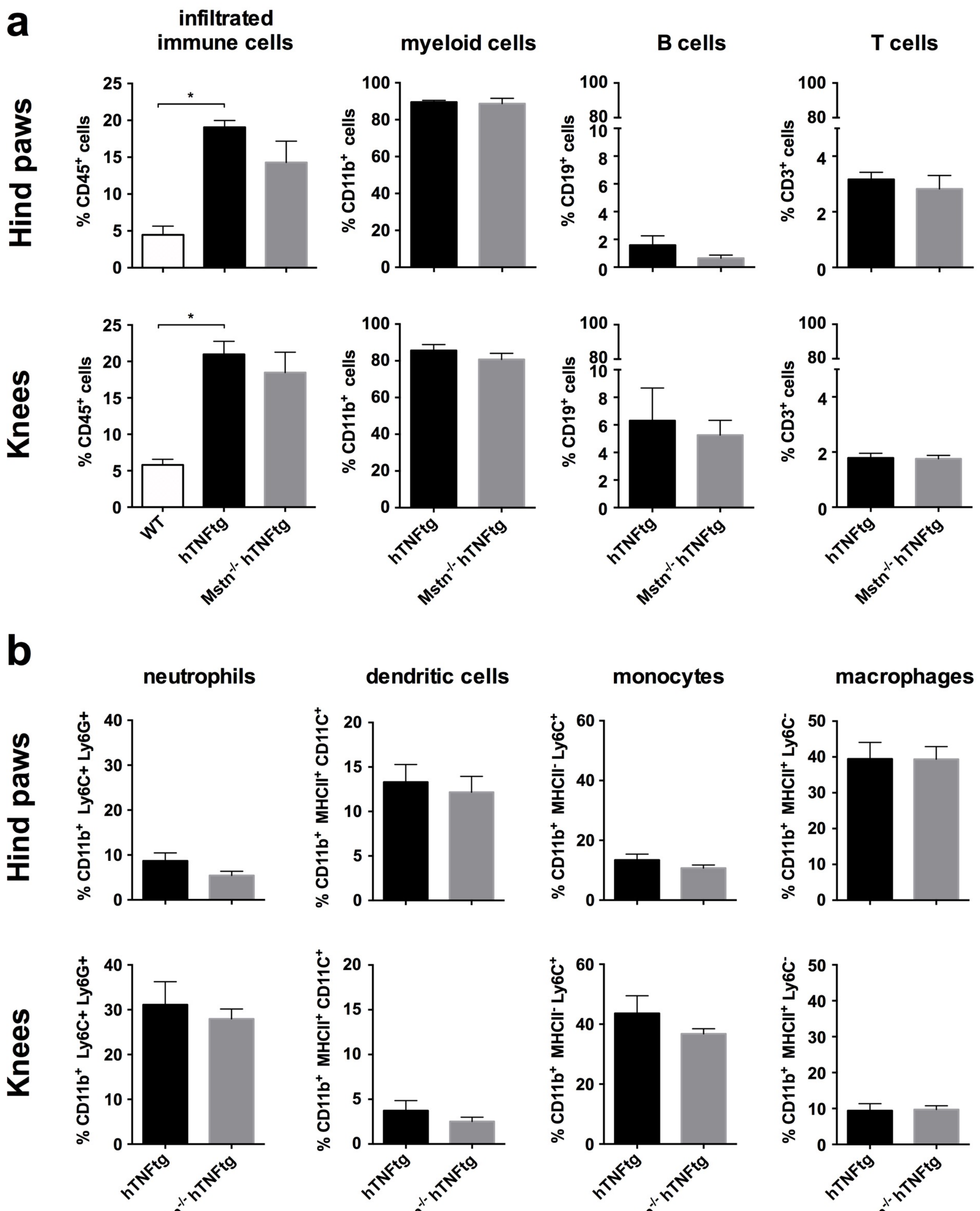

Figure S1: Comparative analysis of infiltrated immune cell populations in arthritic joints

Analysis of inflammatory cell populations in joints of hind paws and knees of WT, hTNFtg, and Mstn$^{-/-}$ hTNFtg mice by flow cytometry. **a** Quantification of leukocytes

($CD45^+$) infiltration in arthritic joints compared to uninflamed WT joints and comparative analysis of infiltration of myeloid cells ($CD11b^+$), B cells ($CD19^+$), and T cells ($CD3^+$) in joint tissues of hTNFtg and $Mstn^{-/-}$ hTNFtg mice. Data represent means ± SEM (WT n = 3, hTNFtg knees n = 6, hind paws n = 7, $Mstn^{-/-}$ hTNFtg n = 5, Mann-Whitney *U* Test, * = $p \leq 0.05$). **b** Subdivision of the myeloid cell population in neutrophils ($CD11b^+$ $Ly6C^+$ $Ly6G^+$), dendritic cells ($CD11b^+$ $MHCII^+$ $CD11c^+$), monocytes ($CD11b^+$ $MHCII^–$ $Ly6C^+$), and macrophages ($CD11b^+$ $MHCII^+$ $Ly6C^–$). Data represent means ± SEM (hTNFtg knees n = 4, hind paws n = 5, $Mstn^{-/-}$ hTNFtg n = 5), Mann-Whitney *U* Test.

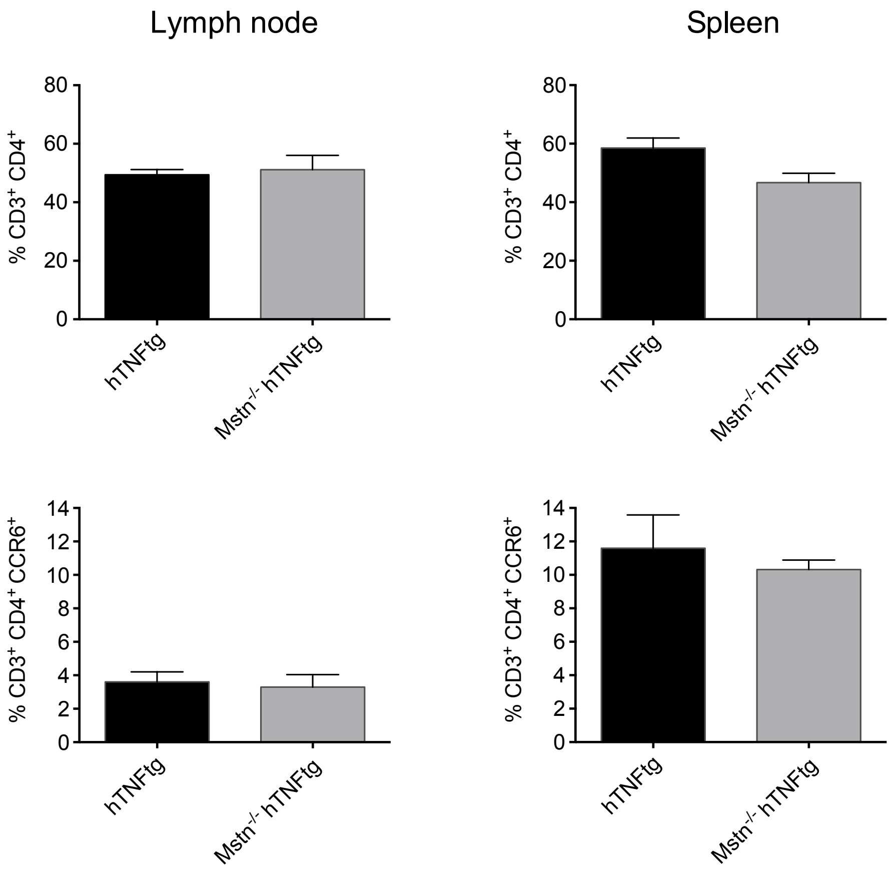

Figure S2: Comparative analysis of Th cell populations in lymph node and spleen

Quantification of Th cells ($CD3^{+}CD4^{+}$) and Th17 cells ($CD3^{+}CD4^{+}CCR6^{+}$) in lymph node and spleen of hTNFtg and $Mstn^{-/-}$ hTNFtg mice by flow cytometry. Data represent means ± SEM (hTNFtg n = 5, $Mstn^{-/-}$ hTNFtg n = 5, Mann-Whitney *U* Test).

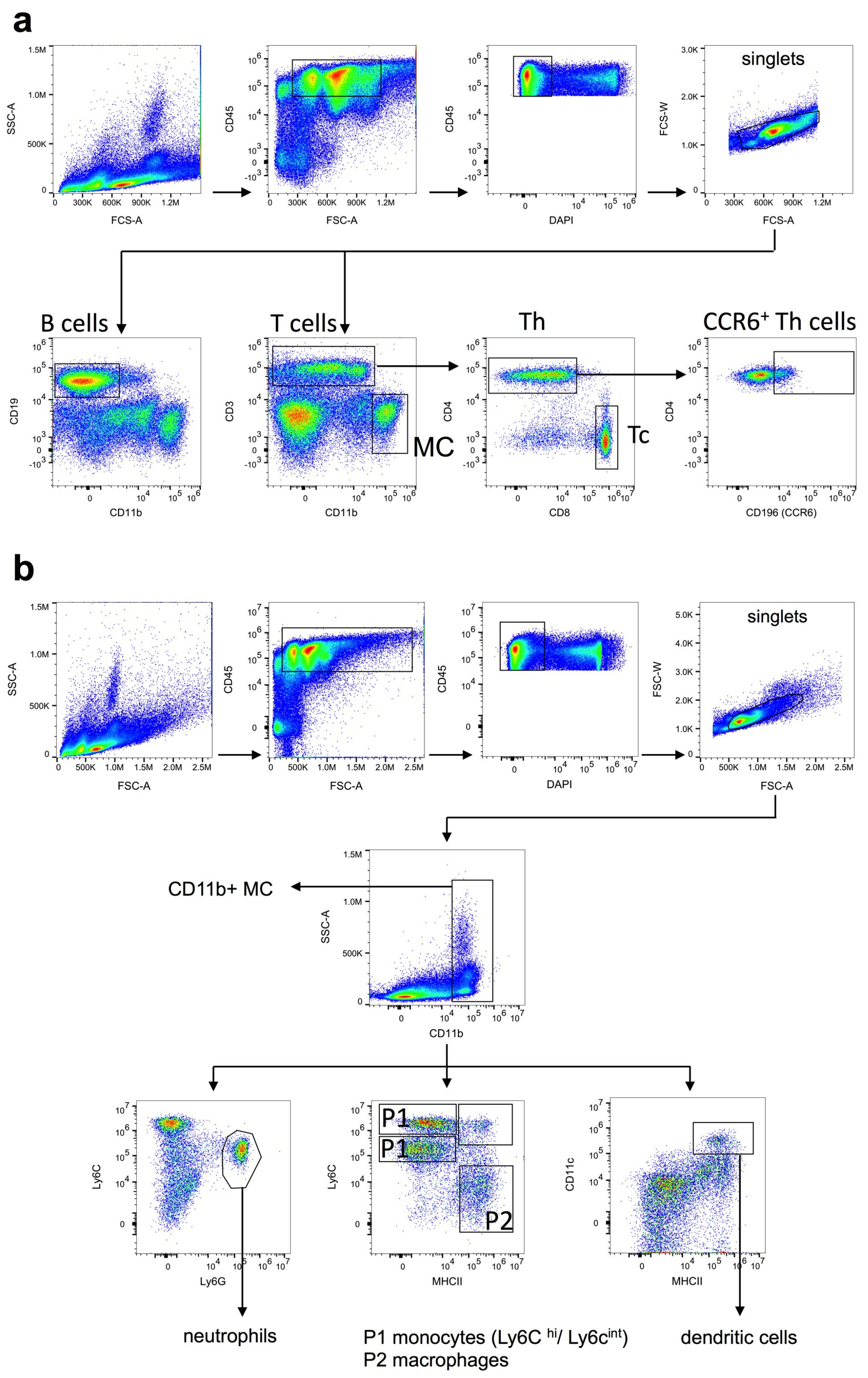

Figure S3: FACS gating strategy to analyse immune cell infiltration of mouse hind paw and knee joints

Gating strategy to analyse the different cellular proportions by FACS. **a** To analyse cell populations derived from the lymphoid lineage, cells were first gated against the size

(FSC-A) and CD45 positive cells. Then, DAPI negative ($CD45^+$ $DAPI^-$) cells were selected to exclude dead cells. Single cells (singlets, $CD45^+$ $DAPI^-$) were selected using FCS-A – FCS-W to exclude doublets. The single cell populations were investigated and gated by the expression of $CD11b^+$ on myeloid cells (MC), $CD19^+$ on B-cells ($CD19^+$ $CD11b^-$), and $CD3^+$ on T-cells ($CD3^+$ $CD11b^-$). The $CD3^+$ T-cell population was subdivided in Th ($CD3^+CD4^+$) and Tc ($CD3^+CD8^+$) cells. In addition, $CD3^+$ $CD4^+$ Th cells were further analysed for the expression of CCR6 ($CD196^+$). **b** Equally to the gating strategy of lymphoid cells, for analysis of cell populations derived from the myeloid lineage, single cell populations were gated by the expression of $CD11b^+$ on myeloid cells ($CD11b^+$ MC) to exclude cells from the lymphoid lineage. The $CD11b^+$ MC population was further investigated and gated by the expression of $CD11c^+$ $MHCII^+$ on DCs and by the expression of $Ly6C^+$ $Ly6G^+$ on neutrophils. Furthermore, the $CD11b^+$ MC population was gated by the expression of $Ly6C^{hi}$ $MHCII^-$ and $Ly6C^{int}$ $MHCII^-$ monocytes (P1), and $Ly6C^-$ $MHCII^+$ macrophages (P2). (tissue: spleen; singlets: single cells, MC: myeloid cells, Th: T helper cells, Tc: T cytotoxic cells).

Full-length unedited blots of Figure 2

Figure 2 e Mstn

pNFkB

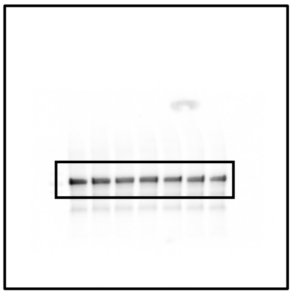

tNFkB

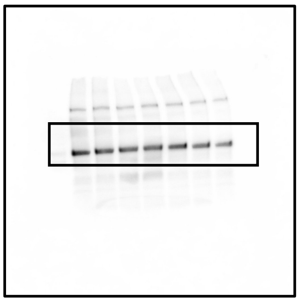

pERK

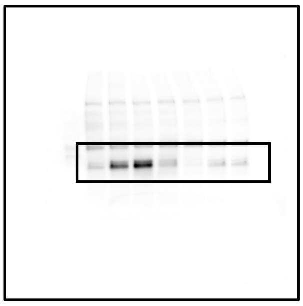

tubulin

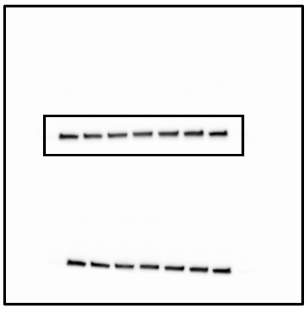

pp38

Low exp

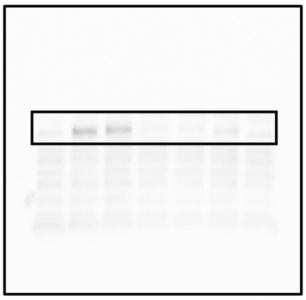

high exp

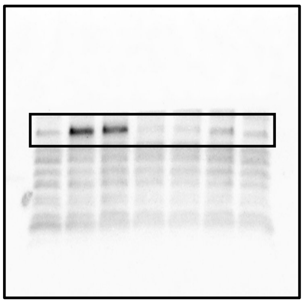

tubulin

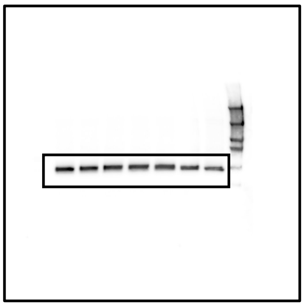

Smad2

Low exp

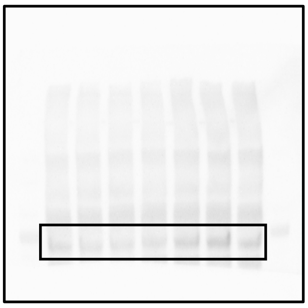

high exp

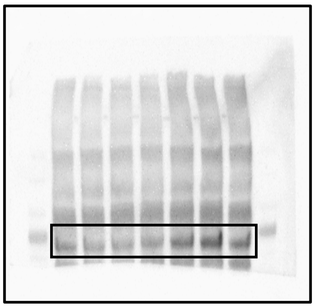

tubulin

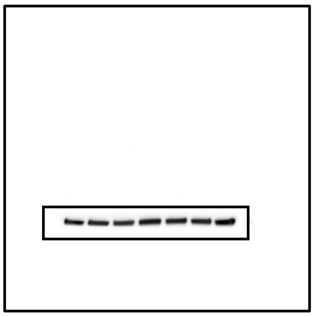

Figure 2 e IL-17A

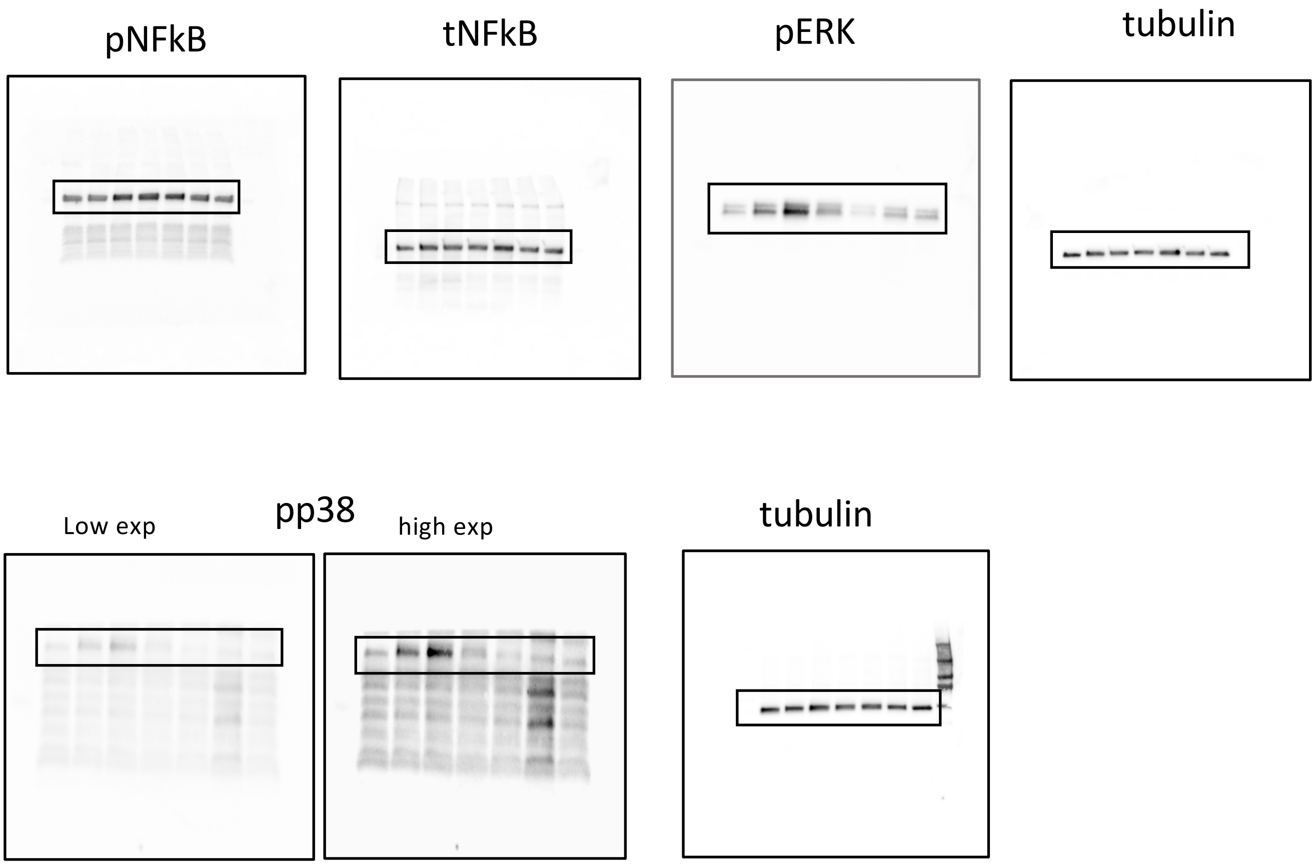

__Figure 2 f__ We are not able to provide original images of lower contrast for Figure 2f-pp38

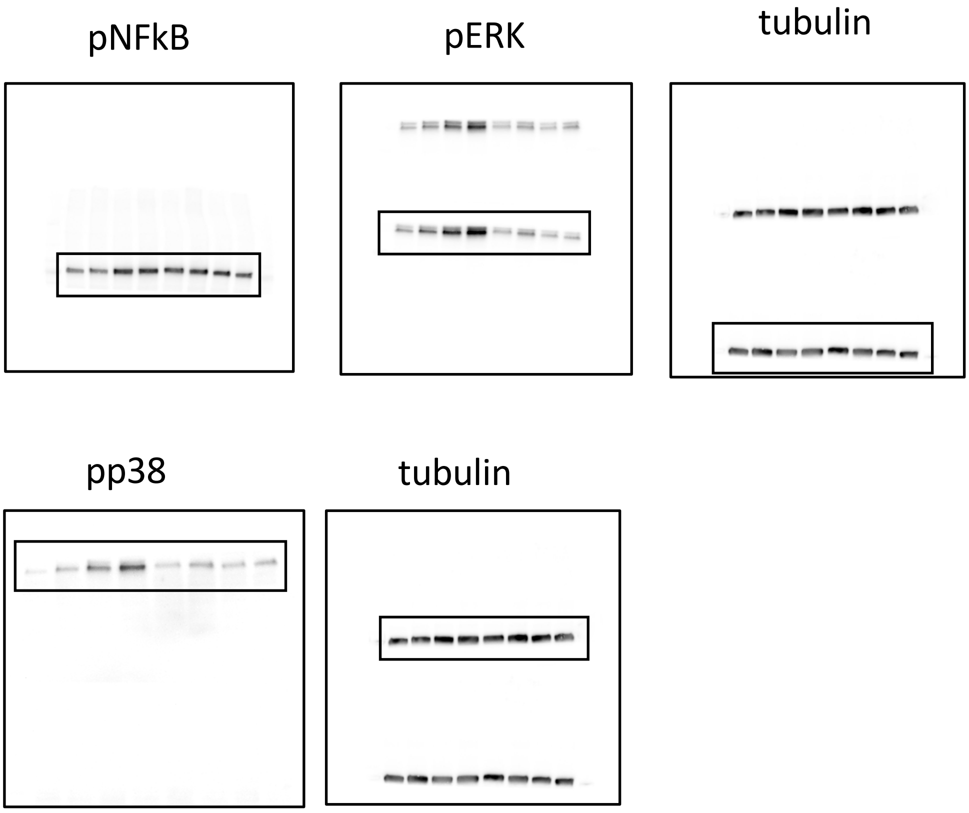

Figure 2 g We are not able to provide original images of lower contrast for Figure 2g-pp38

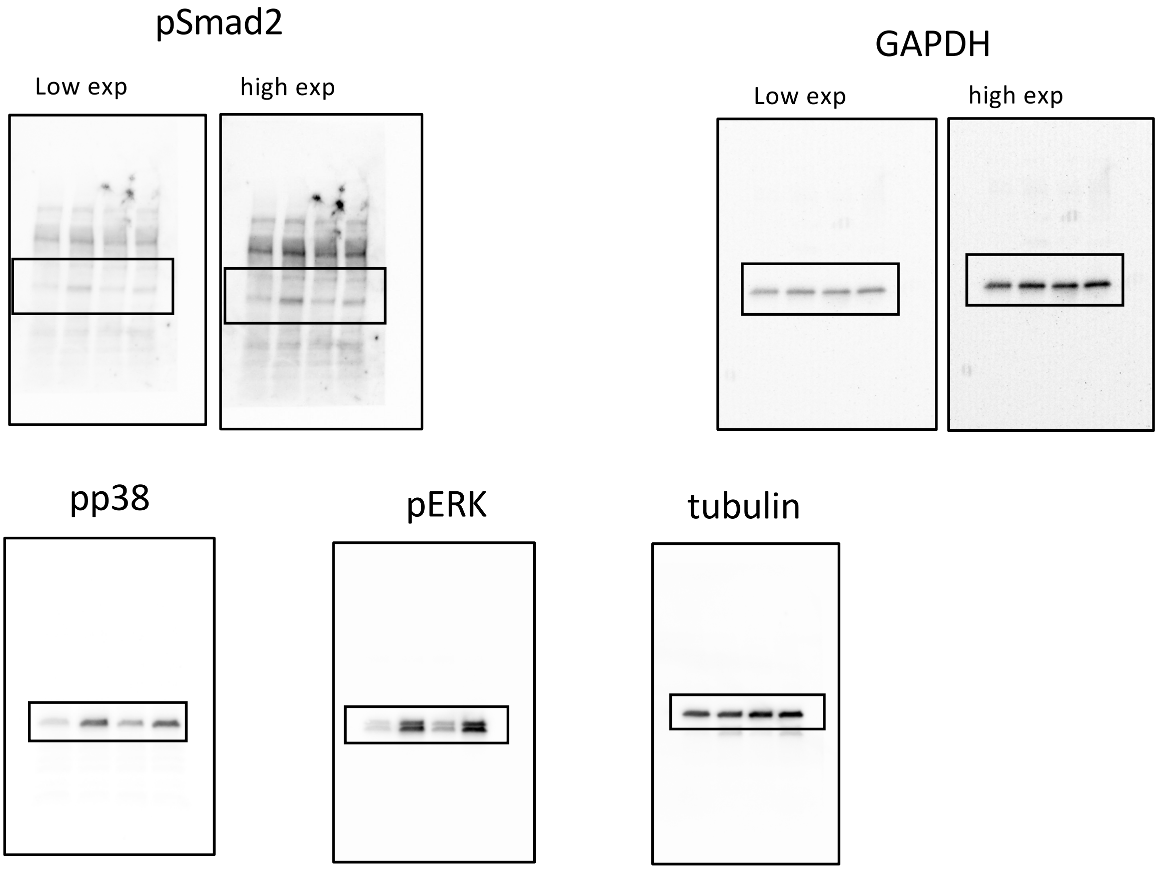

Supplement: Supplementary file 1 — Supplementary Information. [file 41598_2021_93599_MOESM1_ESM.pdf]
